# Outdoor Environmental Risk Factors for Paediatric Ragweed Sensitisation: A Formal Model Comparison

**DOI:** 10.3390/children13101366

**Published:** 2026-10-08

**Authors:** Mario Lovrić, Ivana Banić, Kristina Časni, Iva Šunić, Ivana Jerković, Mirjana Turkalj

**Affiliations:** 1 The Lisbon Council, 1040 Brussels, Belgium; 2 Institute for Anthropological Research, 10000 Zagreb, Croatia; 3 Srebrnjak Children’s Hospital, 10000 Zagreb, Croatia; 4 Know Center Research GmbH, 8010 Graz, Austria; 5 Faculty of Medicine, Josip Juraj Strossmayer University of Osijek, 31000 Osijek, Croatia; 6 Faculty of Medicine, Catholic University of Croatia, 10000 Zagreb, Croatia

**Keywords:** ragweed sensitisation, paediatric allergy, air pollution, green spaces, model selection

## Abstract

**Highlights:**

**What are the main findings?**

- PM_10_ and categorical pollen exposure were statistically equivalent predictors of ragweed sensitisation, consistent with their strong area-level correlation; NO_2_ provided no additional information; indoor exposures added no independent risk.
- Children with more green space within 5 km of home and children living in rural locations were less often sensitised; whether broadleaved forest cover explains the rural association could not be determined.

**What are the implications of the main findings?**

- When two environmental exposures are highly correlated, comparing models by an information criterion shows whether the data can tell them apart at all, which guards against attributing sensitisation risk to one exposure when the two cannot be statistically distinguished.
- The association between surrounding green space and lower paediatric ragweed sensitisation is hypothesis-generating; confirmation in longitudinal studies would be needed before drawing implications for urban greening as a preventive strategy.

**Abstract:**

**Background/Objectives:** Ragweed (*Ambrosia artemisiifolia*) is a major aeroallergen in Central and Eastern Europe. Croatian children in regions with twofold difference in pollen exposure show near-identical sensitisation prevalence (the “Zagreb paradox”), and an earlier analysis found regional pollen and rural residence to be the only associated environmental factors. We re-examined whether air pollution, surrounding landscape and indoor environment contribute to it. **Methods:** This cross-sectional analysis used 3520 of the 4016 children recruited to the EU FP7 ATOPICA study, who had their complete data re-analysed within the EU Horizon Europe EDIAQI project. Sensitisation was defined as a skin prick test wheal of at least 2 mm. Environmental predictors (particulate matter, PM_10_; nitrogen dioxide, NO_2_; regional pollen; CORINE land cover within 5 km; rural residence; and indoor exposures) were compared using the Akaike Information Criterion (AIC) in logistic regression models, which were adjusted for age and parental atopy. **Results:** Sensitisation prevalence was 10.8%. PM_10_ and categorical pollen area fitted the data equally well (ΔAIC 1.7): both describe the same inland-coastal contrast and cannot be distinguished with area-level data. NO_2_ added nothing to either. Of the 16 landscape variables, only transitional woodland-shrub remained associated with sensitisation after Bonferroni correction (adjusted odds ratio, aOR 0.93 [95% confidence interval, CI 0.90–0.97]); total green area was nominally associated (aOR 0.92 [95% CI 0.87–0.98]; *p* = 0.009). Children in rural locations were less often sensitised (aOR 0.69 [95% CI 0.50–0.96]; *p* = 0.026), as previously reported; a mediation analysis could not determine whether broadleaved forest cover explains this association. Indoor exposures added little to model fit. **Conclusions:** Area-level data cannot separate particulate air pollution from pollen exposure, so a contribution of air pollution can be neither confirmed nor excluded. Rural residence and, with less certainty, surrounding green space were associated with less sensitisation, and the indoor environment added little to it.

## 1. Introduction

The rapid range expansion of common ragweed (*Ambrosia artemisiifolia*) across Europe represents a critical escalation in the continent’s aeroallergen burden. As a non-native invasive neophyte originating from North America, this annual weed has aggressively colonised major European regions, including the Pannonian Plain, the Rhône Valley, and Northern Italy [1]. Driven by anthropogenic land-use intensification, habitat disturbances and climate changes, its geographical distribution is projected to continue migrating northward and eastward [2]. Ragweed’s exceptionally high allergenic potential is underscored by its prolific reproductive capacity, with a single mature specimen capable of yielding up to several billion pollen grains per season [3]. Due to their small aerodynamic diameter and low settling velocity, these potent pollen grains frequently utilise strong summer updrafts to achieve long-distance transport, as well as anthropogenic influences such as transport of agricultural products and the transport of contaminated soil to travel several hundred or even thousands of kilometres across national borders [4]. Consequently, these extensive atmospheric plumes trigger severe respiratory symptoms, such as allergic rhinitis and asthma, even in clinical populations residing far from established, heavily infested biological source zones. In Central and Eastern Europe, seasonal ragweed pollen concentrations now rival those of established allergens such as grass and birch pollen, and sensitisation rates in high-exposure and high-risk populations (including children) can exceed 15–20% [5,6]. Croatia occupies a distinctive position within this landscape: the agricultural lowlands of Slavonia produce some of the highest ragweed pollen burdens recorded in Europe, with seasonal integrals exceeding 13,000 grains/m^3^ per year [5,7].

Against this background, Perica et al. [7] described an exposure paradox: children in Zagreb exhibited ragweed sensitisation rates (14.8%) practically indistinguishable from those in Slavonia (14.3%), despite their pollen exposure load being approximately one-third as high. Children on the Mediterranean coast, where pollen counts are negligible (~422 grains/m^3^ per year), exhibited sensitisation rates of only 1.5%. Additionally, there were no differences in de novo ragweed sensitisation rates during three consecutive pollination seasons between the Zagreb and Slavonia regions (2.88% and 2.73%, respectively), while the Mediterranean region reported no newly developed sensitisations [8]. This confirms a strong dose–response relationship between very low and high ragweed exposure levels and sensitisation rates, with a conspicuous failure of that relationship between the two inland regions having medium high to high levels of exposure. These results may tentatively suggest a cut-off threshold in ragweed pollen concentrations required to elicit sensitisation or that other environmental factors (such as urbanicity and air pollution) act synergistically with ragweed pollen to enhance their allergenic potential. However, the mechanisms causing this effect remain unclear. Agnew et al. [5] subsequently conducted the first comprehensive environmental epidemiological analysis of the Croatian “Atopic diseases in changing climate, land use and air quality” (ATOPICA) cohort (n = 4015), screening more than 40 variables including air pollutants (particulate matter with a diameter of 10 µm or less, PM_10_; nitrogen dioxide, NO_2_; sulphur dioxide, SO_2_; ozone, O_3_) and all 44 CORINE (Coordinated Information on the European Environment) Land Cover categories within a 5 km buffer. Pollen area (adjusted odds ratio, aOR 12–16 for the highest-pollen regions versus the Mediterranean) and rural residence (aOR 0.73 [95% confidence interval, CI 0.55–0.98]) were the only modifiable factors associated with sensitisation; all air pollutants and all individual land cover types were non-significant. A complementary longitudinal analysis by Jones et al. [9] similarly found no independent association between daily PM_10_, NO_2_, SO_2_, or O_3_ and symptom days in sensitised Croatian children. Neither study, however, formally compared competing environmental predictors using a framework that accounts for model parsimony.

Two analytic gaps remain. First, the ecological correlation between PM_10_ and pollen is high across the three study regions (child-level r = 0.79; r = 0.84 across the three regional means), driven by the Mediterranean having both the lowest PM_10_ (~16 µg/m^3^) and the lowest pollen (~422 grains/m^3^ per year), while inland Croatia has similarly elevated PM_10_ (~32 µg/m^3^) in both Zagreb and Slavonia regardless of their twofold pollen difference. Models built on different, highly correlated area-level predictors can be compared on a common scale with the Akaike Information Criterion (AIC), which weighs model fit against the number of parameters. Second, while Agnew et al. [5] found all 44 individual CORINE categories non-significant, composite landscape indices and formal mediation analysis to partition the rural protection effect have not been applied to this cohort.

The aim of this study was therefore to examine and revisit the ATOPICA Croatia cohort data of 4016 children in order to assess: (1) whether pollen area and air pollutants provide distinct or informationally equivalent predictions of ragweed sensitisation using AIC-based model comparison; (2) whether composite green-space indicators, including broadleaved forest cover, are independently associated with sensitisation, and whether the apparent protective association of rural residence can be attributed to landscape composition; and (3) whether indoor environmental exposures contribute to sensitisation in a joint environmental model.

## 2. Materials and Methods

### 2.1. Study Design and Participants

**Figure 1:**
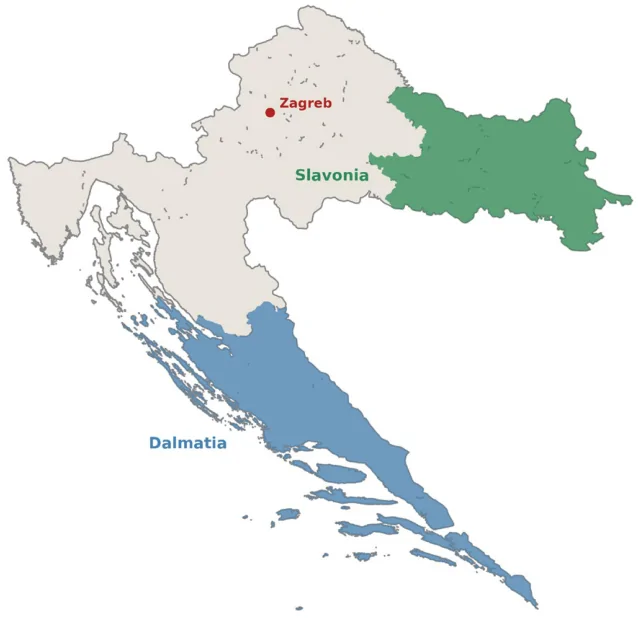
Study area: Slavonia (eastern Croatia), city of Zagreb (central Croatia), and Dalmatia (Adriatic coast).

The ATOPICA (Atopic diseases in changing climate, land use and air quality, grant agreement number: 282687) project is a European, prospective, multi-regional study of environmental determinants of allergic sensitisation in children. The Croatian cohort comprised 4016 children aged 2–13 years, recruited between April 2012 and June 2014 through primary schools and kindergartens in three geographically and ecologically distinct regions: Zagreb, the Mediterranean coast (Dalmatia), and Slavonia (Figure 1). These regions differed in a number of environmental and lifestyle factors, including urbanicity, air pollution and ragweed pollen concentrations. The present analysis is cross-sectional and uses the baseline assessment.

The study was approved by the Local Ethics Committee of Children’s Hospital Srebrnjak, Croatia (on 2 March 2012, Class No 1/2012). Parents or legal guardians provided written informed consent prior to enrolment. The participants were children, both girls and boys, whose parents/caregivers agreed for them to participate in the studies after receiving both oral and written information about the study to their satisfaction those not meeting the criteria for exclusion. Criteria for inclusion and exclusion were assessed by a specialist physician (paediatric allergy specialist with 20+ years of expertise in the field). Criteria for inclusion: children aged 2–13 years, healthy and non-allergic, as well as allergic participants. Exclusion criteria were known malignancies, congenital and other serious chronic illnesses preventing reliable results (e.g., severe metabolic and mast cell disorders, etc.). Furthermore, children were excluded from participation and biomaterial collection in case of a fever of at least 38.5 °C or known acute infection.

### 2.2. Outcome

The primary outcome was ragweed sensitisation, defined as a positive skin prick test (SPT) response to *Ambrosia artemisiifolia* allergen extract (Alyostal ST-IR, Stallergenes Greer International AG, Baar, Switzerland) with a mean wheal diameter of 2 mm or more above the negative control, assessed according to the ISAAC (International Study of Asthma and Allergies in Childhood) standardised protocol [10] and suitable for younger children [11], consistent with Agnew et al. [5].

### 2.3. Environmental Exposures

#### 2.3.1. Air Pollution

Using ArcGIS 10.2 (Esri), each child was assigned long-term (2007–2012) annual mean concentrations of PM_10_, NO_2_, SO_2_, and O_3_ from Airbase V8 (European Environment Agency), assigned at the area level. The regional annual mean PM_10_ was 32.2 µg/m^3^ in Zagreb, 31.7 µg/m^3^ in Slavonia and 15.8 µg/m^3^ in the Mediterranean region; the corresponding NO_2_ means were 32.8, 18.5 and 28.9 µg/m^3^. PM_10_ did not follow a simple urban-rural gradient: both inland regions had substantially higher PM_10_ than the Mediterranean, whereas NO_2_ showed the expected urban hierarchy. Because pollutant exposure was assigned by area, all pollution findings are subject to the ecological fallacy.

#### 2.3.2. Pollen Exposure

Long-term (2007–2012) seasonal ragweed pollen counts were obtained from eight stations of the Croatian Environment Agency. Children were grouped into four pollen areas: Slavonski Brod (~13,039), Osijek (~8275), Zagreb (~5189), and Mediterranean (~422 grains/m^3^ per year). Because Slavonski Brod and Osijek are both located within the Slavonia region and had the two highest pollen burdens, these two stations were combined into a single “Slavonia” category for all regression and AIC analyses, yielding the three-level regional variable (Zagreb, Mediterranean, Slavonia) used throughout the statistical analysis and in Table 1. The child-level PM_10_-pollen correlation was r = 0.79 in the analytic sample; across the three regional means it was r = 0.84. The two Slavonian areas did not differ in sensitisation (likelihood-ratio χ^2^ = 1.15, 1 degree of freedom; *p* = 0.283).

#### 2.3.3. Land Cover and Landscape Composition

Land cover areas for all 44 CORINE Land Cover 2006 categories were calculated within a 5 km buffer around each child’s home using ArcGIS 10.2. All areas were log-transformed prior to analysis (natural log of the area in m^2^ plus 1, so that zero areas are retained; 19% of children had no broadleaved forest and 33% no pasture within 5 km). Supplementary Table S4 also gives odds ratios per doubling of area. Three composite indices were defined as fixed groupings of CORINE categories rather than being assembled from the results of the univariate screening reported in Section 3.3: (1) total green area (the summed area of broadleaved, coniferous and mixed forest, natural grasslands and pastures); (2) urban area (the summed area of continuous and discontinuous urban fabric, industrial or commercial units, and road and rail networks); and (3) the Shannon landscape diversity index H = -sum(π ln[π]) across the 11 land cover classes that were screened. Rural or urban residence was taken from the ATOPICA dataset as classified by Agnew et al. [5]: home locations that intersected CORINE “artificial surfaces” were coded urban and all others rural.

Indoor exposures were ascertained by the ISAAC Phase II parental questionnaire: environmental tobacco smoke (ETS), pet ownership, carpet, mattress, type of heating source and feather-filled bedding.

### 2.4. Statistical Analysis

As part of the retrospective data analysis within the EU Horizon Europe EDIAQI (Evidence-driven indoor air quality improvement, grant agreement number: 101057497) clinical, environmental and other data were analysed using binary logistic regression models. This is a hypothesis-generating secondary analysis of previously collected cohort data; no formal statistical analysis plan was pre-registered for this specific re-analysis.

These binary logistic regression models were fitted by maximum likelihood estimation using Python 3.11 (statsmodels 0.14 [12]). Complete cases were used, defined as children with data on the outcome, study region, age, parental atopy, land cover, rural residence and the four indoor exposures; missing data were not imputed. Results are reported as aOR with 95% confidence intervals (95% CI) and exact *p*-values. A 2-sided significance level of 0.05 was used throughout. For analyses using PM_10_ as a binary exposure, children were classified as “high PM_10_” if their area-level annual mean was at or above the 66th percentile of the analytic sample’s PM_10_ distribution; because area-level PM_10_ takes only four distinct values in this cohort, any cut-off between the two Mediterranean and the two inland values gives the same split, so binary PM_10_ is identical to an inland (Zagreb, Slavonia) versus Mediterranean indicator and classified 60.1% of children as high-exposure. The PM_10_-only model is therefore the pollen-area model with Zagreb and Slavonia constrained to be equal. An alternative median-split threshold is examined in the Supplementary sensitivity analyses (Table S2a,b). Because children were recruited through schools and exposures were assigned by area, we repeated the rural, landscape and joint models with standard errors clustered on school (105 schools; school recorded for 3431 children) and with additional adjustment for sex, which was recorded for 3198 children (Supplementary Table S5).

We used the Akaike Information Criterion (AIC = 2k − 2 ln L-hat) to formally compare six candidate models: base (age and parental atopy), pollen area (3-category), PM_10_-only, NO_2_-only, PM_10_ + NO_2_, and NO_2_ + pollen area. Models that combine binary PM_10_ with pollen area were not candidates, because binary PM_10_ is fully determined by region and would add a redundant term. Differences of fewer than 2 AIC points are considered negligible; differences greater than 10 indicate strong evidence in favour of the lower-AIC model [13].

Sixteen landscape variables were screened individually (11 CORINE land cover classes, the distance to the nearest road and to the nearest motorway, and the three composite indices; Supplementary Table S4), each adjusted for age, parental atopy, rural residence, and study region (Bonferroni-corrected threshold *p* < 0.003).

Because odds ratios are non-collapsible, comparing logistic regression coefficients across models (the Baron–Kenny approach) is not a statistically valid way to quantify mediation of a binary outcome. We therefore used a parametric counterfactual mediation analysis to test whether broadleaved forest cover mediated the association between rural residence and sensitisation, estimating the total, natural direct and natural indirect effects on the risk (probability) scale, with 95% confidence intervals from a 2000-replicate nonparametric bootstrap. The mediator model was a linear regression of log broadleaved forest area on rural residence, and the outcome model a logistic regression of sensitisation on rural residence, log broadleaved forest area and their interaction; both were adjusted for study region, age and parental atopy.

Four model families were compared by AIC: base; indoor-only (ETS, pet, carpet, feather plus base covariates); outdoor-only (PM_10_, log-broadleaved forest, log-pastures, rural residence, base covariates); and combined.

## 3. Results

### 3.1. Participants

**Figure 2:**
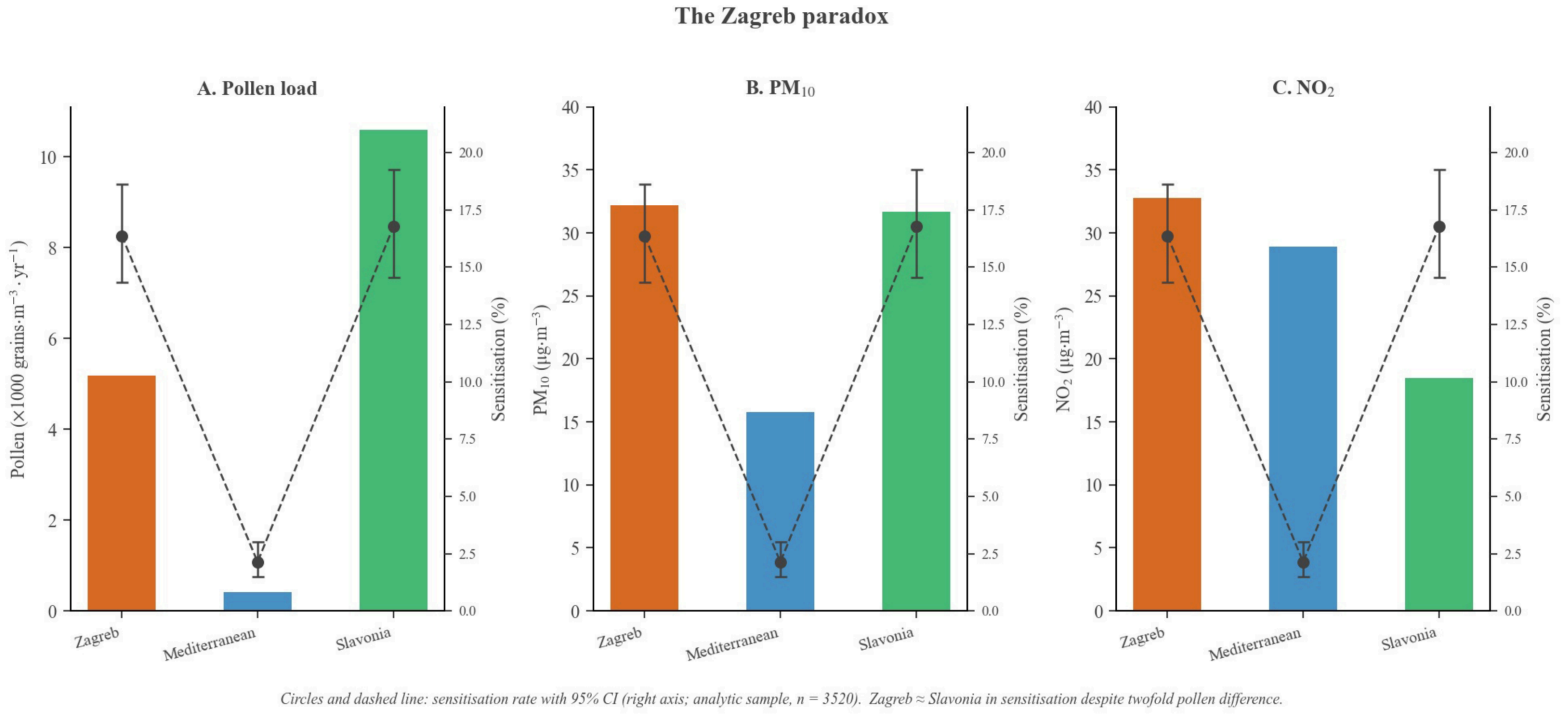
The Zagreb paradox. Ragweed sensitisation rates by study region in the analytic sample (n = 3520; circles, right axis, with 95% confidence intervals) plotted against (**A**) long-term annual mean pollen exposure, (**B**) PM_10_ and (**C**) NO_2_ (bars, left axis). Zagreb and Slavonia show nearly identical sensitisation rates (16.4% and 16.8%) despite a twofold difference in pollen burden.

A total of 4016 children were enrolled; 3520 (87.7%) had complete data for the primary analysis. Of these, 380 (10.8%) were ragweed-sensitised. Sensitisation rates differed markedly by study region: 16.4% in Zagreb (n = 1142), 2.1% in the Mediterranean region (n = 1406), and 16.8% in Slavonia (n = 972). The mean age was 7.0 years (standard deviation, SD 1.8; range 2–13 years), and parental atopy was reported in 38.2% of families; 51.1% of the 3198 children with recorded sex were boys, and 440 children (12.5%) lived in rural locations (Supplementary Table S7). Sensitisation rates in Zagreb (16.4%) and Slavonia (16.8%) were nearly identical despite Slavonia’s twofold higher pollen burden, consistent with the Zagreb paradox [7]. Zagreb’s NO_2_ concentration was 14.3 µg/m^3^ higher than Slavonia’s, while PM_10_ was essentially equal (difference 0.5 µg/m^3^; Figure 2). Children excluded from the primary analysis because of missing data (n = 496) differed modestly from those included in regional composition (33.1% vs. 39.9% Mediterranean, 36.7% vs. 32.4% Zagreb, 30.2% vs. 27.6% Slavonia; χ^2^ = 8.72, *p* = 0.013), but not in age (7.12 vs. 6.98 years; *p* = 0.191; recorded for 364 of the excluded children), parental atopy (34.0% vs. 38.2%; *p* = 0.453; recorded for 100 of the excluded children), or sensitisation status (8.9% vs. 10.8%; *p* = 0.220).

### 3.2. AIC Model Comparison: Pollen vs. Air Pollution

Six candidate models were compared by AIC (Table 1). The three top-performing models were PM_10_ only (AIC = 2157.1), pollen area 3-category (AIC = 2158.8, ΔAIC = 1.7), and PM_10_ + NO_2_ (AIC = 2158.8, ΔAIC = 1.7). All three outperformed the base model by 216 to 218 AIC points. Binary PM_10_ cannot add to the pollen-area model because it is fully determined by region, and adding NO_2_ to the pollen area did not improve fit (ΔAIC = 3.7). The 1.7-point difference between the PM_10_-only and pollen-area models corresponds to the contrast between Zagreb and Slavonia (likelihood-ratio χ^2^ = 0.33, 1 degree of freedom; *p* = 0.567). In the single-predictor models, high PM_10_ had an aOR of 9.13 [95% CI 6.24–13.36]; in the pollen-area model, relative to Zagreb, the Mediterranean region had an aOR of 0.11 [95% CI 0.08–0.17] and Slavonia an aOR of 1.07 [95% CI 0.85–1.35]. This near-identical fit is consistent with the high area-level collinearity between PM_10_ and pollen (Section 2.3.2) and should be read as AIC confirming that the two exposures are not statistically distinguishable in this ecological design, rather than as AIC identifying which of the two is the true driver of sensitisation. NO_2_ alone improved only modestly on the base model (AIC 6.8 points lower) and added nothing to PM_10_ or pollen area (AIC 210.8 points above the best model). When pollen was modelled as a continuous variable, it performed substantially worse than PM_10_ (ΔAIC = 107.8; continuous pollen AIC = 2266.1 vs. continuous PM_10_ AIC = 2158.3). This reflects a modelling artefact: Zagreb and Slavonia show virtually identical sensitisation rates despite a twofold pollen difference. When pollen was modelled categorically, its predictive performance was indistinguishable from PM_10_ (ΔAIC = 1.7).

### 3.3. Landscape Composition and Green Space

**Figure 3:**
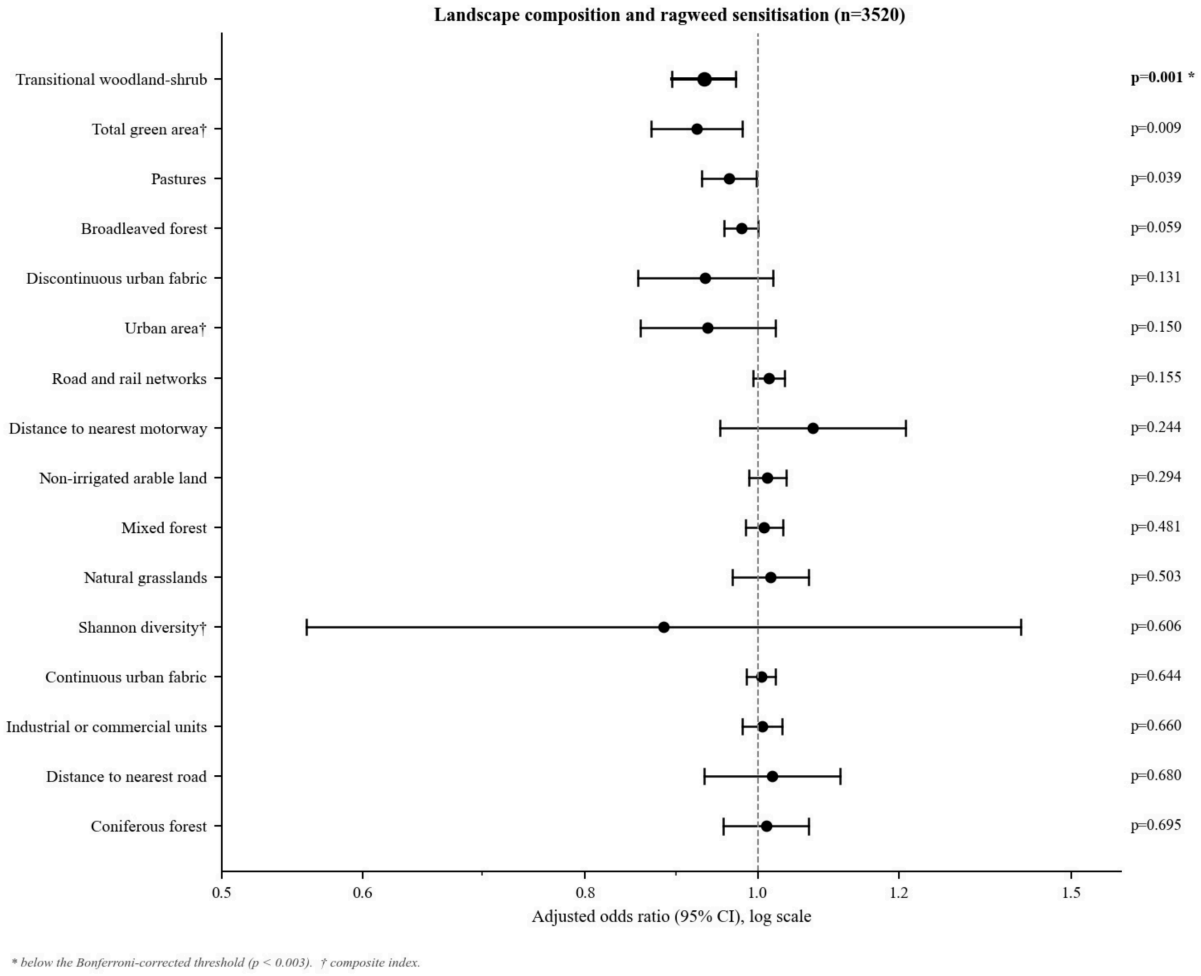
Forest plot of the sixteen landscape variables in relation to ragweed sensitisation (n = 3520). Adjusted odds ratios (aOR) and 95% CI per log-unit increase in area or distance (per unit for the Shannon index), adjusted for age, parental atopy, rural residence, and study region. Bonferroni-corrected significance threshold: *p* < 0.003.

All sixteen screening results are shown in Figure 3 and Supplementary Table S4. One variable exceeded the Bonferroni-corrected threshold (*p* < 0.003): transitional woodland-shrub area (aOR 0.933 [95% CI 0.895–0.972]; *p* = 0.001). Three further variables showed nominal associations in the same direction: total green area (aOR 0.92 [95% CI 0.87–0.98]; *p* = 0.009), pasture area (aOR 0.963 [95% CI 0.930–0.998]; *p* = 0.039) and, borderline, broadleaved forest area (aOR 0.979 [95% CI 0.957–1.001]; *p* = 0.059). Road and rail network area, the urban composite and Shannon landscape diversity were not associated with sensitisation (*p* = 0.155, 0.150 and 0.606, respectively). Children with more forest, shrub and grassland cover within 5 km thus had lower odds of sensitisation independently of their study region and of rural or urban residence, although only the association with transitional woodland-shrub withstood correction for multiple testing.

### 3.4. Rural Residence and Broadleaved Forest Cover

Rural residence was associated with lower odds of ragweed sensitisation after adjustment for study region, age and parental atopy (aOR 0.69 [95% CI 0.50–0.96]; *p* = 0.026), in line with the estimate reported for this cohort by Agnew et al. [5]. Rural homes had considerably more broadleaved forest within 5 km (regression coefficient 2.42 log-units [95% CI 1.86–2.97]; *p* < 0.001), so we asked whether forest cover accounts for the rural association. When broadleaved forest cover was added to the model, the rural estimate changed little (aOR 0.72 [95% CI 0.52–0.99]; *p* = 0.046) and the forest estimate was borderline (aOR 0.979 [95% CI 0.957–1.001]; *p* = 0.059). In the counterfactual mediation analysis on the risk-difference scale (2000-replicate bootstrap), the total effect of rural residence was −3.07 percentage points [95% CI −5.41 to −0.47], but it could not be split reliably into a natural direct effect (−1.42 percentage points [95% CI −6.82 to 2.77]) and a natural indirect effect through forest cover (−1.65 percentage points [95% CI −4.50 to 3.25]); the proportion mediated was correspondingly imprecise (54% [95% CI −165% to 358%]). We therefore do not report a mediation percentage. The rural association was similar with standard errors clustered on school (aOR 0.68 [95% CI 0.52–0.88]; *p* = 0.004; n = 3431, with rural children in 53 of 105 schools) and after additional adjustment for sex (aOR 0.68 [95% CI 0.49–0.94]; *p* = 0.021; Supplementary Table S5).

### 3.5. Indoor Environment

Indoor exposure prevalence did not differ between sensitised and non-sensitised children (Table 2). In a model including all four indoor exposures together with age and parental atopy, aORs ranged from 0.79 (ETS [95% CI 0.61–1.02]; *p* = 0.066) to 1.00 (pet [95% CI 0.76–1.31]; *p* = 0.984), none statistically significant.

Indoor exposures added little to model fit (Table 3). The indoor-only model (AIC = 2377.4) performed worse than the base model (AIC = 2374.7), indicating that the four indoor variables did not repay the complexity penalty (likelihood-ratio χ^2^ = 5.3, 4 degrees of freedom; *p* = 0.256). Adding indoor variables to the outdoor model improved AIC by only 1.2 points (likelihood-ratio χ^2^ = 9.2, 4 degrees of freedom; *p* = 0.056); in that model ETS had aOR 0.77 [95% CI 0.59–1.00]; *p* = 0.054.

### 3.6. Joint Environmental Model

**Figure 4:**
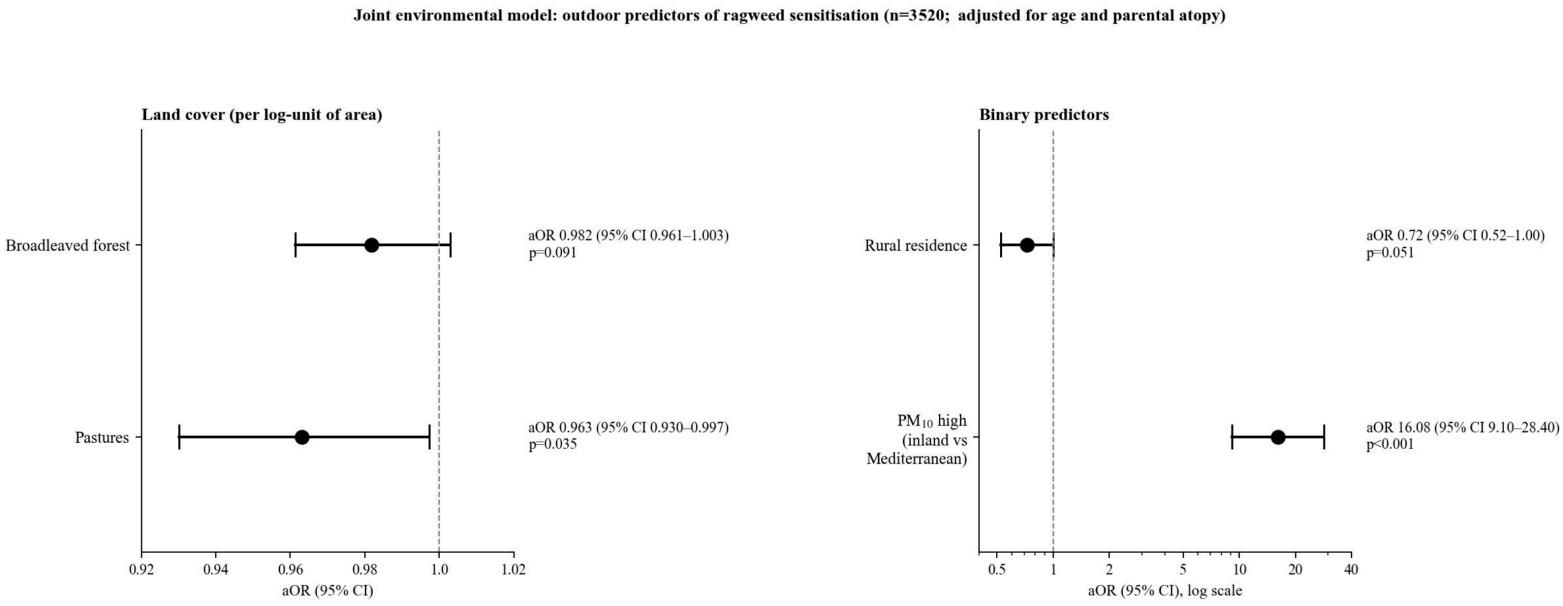
Joint environmental model. Adjusted odds ratios (aOR) and 95% CI for broadleaved forest and pastures (per log-unit of area; **left**) and for rural residence and PM_10_ (binary, inland versus Mediterranean; **right**, log scale) in the joint outdoor model (n = 3520), adjusted for age and parental atopy.

In a joint outdoor model including PM_10_ (binary), log-broadleaved forest (natural log of forest area in m^2^ plus 1) and log-pastures (natural log of pasture area in m^2^ plus 1), plus rural residence, age, and parental atopy (n = 3520; AIC = 2151.7; Table 3’s “Outdoor” model), high PM_10_ was strongly associated with sensitisation (aOR 16.08 [95% CI 9.10–28.40]; *p* < 0.001). This large odds ratio predominantly reflects the inland-Mediterranean contrast rather than a within-region PM_10_ dose–response: only 30 of 1406 children (2.1%) in the Mediterranean region were sensitised, so this stratum contributes limited statistical power and the joint-model PM_10_ estimate should be interpreted as an area-contrast effect rather than a graded exposure-response relationship. Pasture area was associated with lower sensitisation (aOR 0.963 [95% CI 0.930–0.997]; *p* = 0.035), broadleaved forest cover showed a weaker trend (aOR 0.982 [95% CI 0.961–1.003]; *p* = 0.091), and the estimate for rural residence was close to that in Section 3.4 (aOR 0.72 [95% CI 0.52–1.00]; *p* = 0.051). The joint model improved AIC by 5.4 points over PM_10_ alone (2151.7 vs. 2157.1; likelihood-ratio χ^2^ = 11.4, 3 degrees of freedom; *p* = 0.010), so landscape and rural residence carry information beyond the regional contrast (Figure 4).

## 4. Discussion

This study re-examined environmental determinants of ragweed sensitisation in the ATOPICA Croatia cohort using an information-theoretic framework to compare several environmental influences rather than relying solely on statistical significance in multivariable regression models. There were four principal findings:

1. PM_10_, categorical pollen exposure, and the combined PM_10_ + NO_2_ model demonstrated essentially equivalent explanatory performance (ΔAIC ≤ 1.7), indicating that these models contain comparable information regarding ragweed sensitisation.
2. Rural residence was associated with lower sensitisation, as previously reported for this cohort, and the data could not determine whether broadleaved forest cover explains this association.
3. Total green area within a 5 km radius was nominally associated with a lower likelihood of ragweed sensitisation after adjustment for demographic and geographic factors.
4. Commonly reported indoor environmental exposures seemed to contribute little to model performance, supporting the hypothesis that sensitisation to ragweed is determined predominantly by outdoor environmental conditions.

The finding that PM_10_ and categorical pollen exposure were statistically indistinguishable in terms of model performance qualifies the interpretation of previous analyses from this cohort. Agnew et al. [5] identified ragweed pollen as the dominant environmental predictor of sensitisation, while air pollutants did not retain statistical significance after adjustment for pollen exposure. Rather than contradicting those findings, the present analysis shows why they could hardly have been otherwise: with exposures assigned by area, PM_10_ carries almost no information beyond the regional contrast. Agnew et al. [5] noted this limitation themselves when they wrote that the variability of air pollution in the sample was low. When highly correlated environmental exposures are entered simultaneously into a conventional regression model, the resulting coefficients estimate the independent contribution of each exposure after accounting for the others. Under these circumstances, shared explanatory information is effectively partitioned between correlated predictors, increasing standard errors and reducing statistical significance. Hence, a non-significant result in the regression analysis cannot be interpreted as firm evidence that an exposure lacks biological relevance.

On the other hand, an information-theoretic approach addresses the question of whether models based on PM_10_ or pollen provide different amounts of information about the observed data. The finding that the PM_10_ and categorical pollen models differed by less than 2 AIC indicates that neither model is meaningfully better supported than the other. This distinction is important because it shifts the interpretation from identifying a single, predominant environmental predictor towards recognising that multiple correlated environmental exposures may represent the same underlying environmental gradient.

These analyses also bear on the “Zagreb paradox.” Despite approximately twofold differences in long-term ragweed pollen exposure, children living in Zagreb and Slavonia exhibited almost identical prevalence of ragweed sensitisation. This observation is inconsistent with a simple linear dose–response relationship in which progressively greater pollen exposure produces proportionately higher sensitisation rates. Instead, the data suggest either that sensitisation reaches a plateau once pollen concentrations exceed a critical threshold or that other environmental factors modify the effect of pollen exposure. Agnew et al. likewise concluded that sensitisation had reached a threshold in the areas with medium to high pollen levels [5]. NO_2_, which was 14.3 µg/m^3^ higher in Zagreb than in Slavonia, added nothing to the models, which argues against traffic-related pollution as the explanation; PM_10_ was equal in the two inland regions and therefore remains compatible with either explanation. More importantly, this paradox also explains why modelling pollen as a continuous variable performed substantially worse than categorical pollen exposure. Continuous modelling implicitly assumes that each incremental increase in pollen concentration has a similar effect on sensitisation risk across the entire exposure range. In contrast, categorisation accommodates the apparent threshold behaviour observed in this cohort and therefore describes the data better.

The findings of this study are also consistent with the subsequent longitudinal investigation by Jones et al. [9], who found that daily PM_10_, NO_2_, SO_2_ and O_3_ concentrations did not independently predict day-to-day allergic symptoms among children who were already sensitised to ragweed after accounting for pollen concentrations. Although symptom exacerbation and allergic sensitisation represent distinct biological processes, a number of studies suggest that separating the independent effects of pollen and ambient air pollution is challenging because these exposures frequently coexist within the same environments [14,15]. Our findings therefore refine rather than overturn previous conclusions from the ATOPICA study. Agnew et al. [5] correctly demonstrated that ragweed pollen is a major determinant of sensitisation, but the present analysis indicates that particulate pollution captures very much the same environmental information when considered as an alternative explanatory model. The implication is not that PM_10_ replaces pollen as the causal exposure, nor that pollen becomes unimportant. The results of this study suggest that the available observational data cannot reliably distinguish between these competing environmental explanations because both variables appear to characterise a common environmental exposure profile. This distinction has important implications for future epidemiological studies, as it emphasises the need to evaluate competing environmental hypotheses using analytical approaches that explicitly acknowledge model uncertainty and correlated exposures rather than relying exclusively on null-hypothesis significance testing [15].

Rather than representing mutually exclusive influences, particulate and pollen exposures likely act through interconnected pathways. These pathways influence both allergen exposure and host immune responses [16]. Increasing evidence suggests that particulate air pollution modifies not only the concentration of airborne allergens but also their biological activity, making it increasingly difficult to separate the effects of pollen from those of the surrounding atmospheric environment [17]. Experimental studies have demonstrated that particulate matter and gaseous pollutants can alter pollen morphology, increase pollen fragility, promote the release of allergenic proteins and sub-pollen particles, and enhance allergen bioavailability through oxidative modification and nitration of pollen proteins. Pollutants may also adhere directly to pollen grains, creating “polluen” complexes with enhanced inflammatory potential [18]. Moreover, particulate matter induces oxidative stress within the airway epithelium, disrupts epithelial barrier integrity, activates innate immune signalling pathways, and facilitates antigen uptake by dendritic cells, thereby promoting Th2-polarised immune responses characteristic of allergic disease [19].

The lack of an independent predictive value for NO_2_ is also noteworthy. Nitrogen dioxide has frequently been associated with asthma exacerbations and impaired respiratory health, but its role in the development of allergic sensitisation has been less consistent than that of particulate matter [20]. In this study, adding NO_2_ to the PM_10_ model produced negligible improvement in model fit, suggesting that NO_2_ contributes little additional information once the broader outdoor exposure profile is accounted for. However, this finding does not exclude a biological role for NO_x_ but instead suggests that, within this paediatric cohort, NO_2_ alone is unlikely to explain regional differences in ragweed sensitisation. The observation further illustrates the importance of distinguishing between predictors of sensitisation and predictors of symptom severity, as environmental factors influencing immune priming and modulation may differ from those driving specific clinical manifestations [21].

We also found that children living in areas with greater total green cover had lower odds of sensitisation at the nominal significance level, and that pasture and transitional woodland-shrub cover showed associations in the same direction. Rural residence was also associated with lower sensitisation, as in the earlier analysis of this cohort [5], but the mediation analysis could not determine how much of the rural association runs through forest cover. Agnew et al. found no land cover class associated with sensitisation in models with categorised exposures [5]; with area on a continuous log scale, transitional woodland-shrub was the only class that withstood correction for multiple testing here. This finding is consistent with the biodiversity/hygiene hypothesis, which proposes that regular exposure to diverse natural environments enriches the human microbiome, promotes immune regulation and increases immunological tolerance [22,23]. Evidence suggests that reduced contact with environmental microbial diversity in increasingly urbanised settings contributes to immune dysregulation and the rising prevalence of allergic disease [24,25,26].

Our results extend this concept by suggesting that the vegetation surrounding the home is associated with sensitisation in addition to rural residence itself. Forests and other semi-natural vegetation support complex microbial ecosystems that differ substantially from those associated with intensively managed agricultural landscapes or highly urbanised environments [27]. Such landscapes may provide repeated exposure to environmental microorganisms that promote immune maturation during childhood and reduce exposure to disturbed habitats in which ragweed is abundant.

The absence of meaningful associations between common indoor exposures and ragweed sensitisation in this study provides further support for the predominance of outdoor environmental determinants in the case of ragweed sensitisation. Although this study did not cover all indoor determinants (such as ventilation), most of the indoor exposures, including environmental tobacco smoke, pet ownership, carpets and feather bedding, were individually uninformative. Heating source and mattress age were also collected via the ISAAC Phase II questionnaire and examined in exploratory analyses (Supplementary Table S6). Heating type differed strongly between regions, so these models were adjusted for study region; wood heating (aOR 0.75 [95% CI 0.57–0.99]; *p* = 0.043) and a mattress older than three years (aOR 0.76 [95% CI 0.61–0.96]; *p* = 0.020) showed nominal inverse associations, and the other heating and mattress variables showed none. More importantly, these indoor pollutants also collectively failed to improve model performance in predicting ragweed sensitisation. However, these findings should not be interpreted as evidence that indoor exposures are unimportant for allergic disease generally, as indoor allergens remain major determinants of sensitisation to house dust mites, animal dander and moulds. They may simply suggest that sensitisation to ragweed, a predominantly outdoor aeroallergen, is driven primarily by environmental exposures occurring outside the home.

The informational equivalence of PM_10_ and pollen (Section 3.2) should not be interpreted as evidence that the two exposures are biologically interchangeable; future longitudinal studies with repeated exposure assessment and mechanistic biomarkers are needed to clarify causal pathways.

## 5. Strengths and Limitations

The findings of this study should be interpreted in light of both its strengths and limitations. The study is strengthened by the large, population-based cohort with standardised SPT, objective and long-term air quality and pollen data from national monitoring networks, comprehensive GIS-derived land cover data, and formal information-theoretic model comparison. Composite landscape indices extend previous work in this cohort.

Several important limitations apply. First, the pollutant and pollen measurements are ecological (area-level), providing only three exposure contrasts and introducing the potential for ecological fallacy. The three-region structure means that binary PM_10_ coincides with the regional pollen contrast, so no analysis of these data can resolve which is the causal driver, and the confidence intervals of area-level exposures overstate their precision. Secondly, the cross-sectional design precludes assessment of temporality. Exposure estimates were assigned using residential location and therefore cannot capture individual variation in daily activity patterns, school attendance, residential mobility or personal exposure histories. Thirdly, land cover data (CORINE 2006) predate study recruitment by six to eight years and may introduce bias. Moreover, sensitisation to ragweed was defined as a positive SPT result to ragweed extract, which is less specific than specific IgE to ragweed or its components (such as Amb a1). Cross-reactivity with other weeds, especially mugwort, is common in SPT to ragweed and may introduce bias in outcome definition [28]. Additionally, our analyses included only children with complete data. Included and excluded children differed slightly by region, but not in age, parental atopy, or sensitisation status (Section 3.1), suggesting a small potential for bias due to missing data. Finally, unmeasured confounders, particularly socioeconomic status, and other environmental (such as other pollutants, including indoor PM, volatile organic compounds—VOC, polycyclic aromatic hydrocarbons—PAH, etc.) and lifestyle factors (such as dietary habits) may explain residual associations. Although a subset of children had longitudinal follow-up with data on de novo ragweed sensitisation across three pollen seasons [8], the present analysis was restricted to baseline SPT status and could not directly model the temporal sequence between environmental exposure and incident sensitisation. Although rural residence was associated with lower sensitisation (Section 3.4), its total effect could not be split reliably into direct and forest-mediated components, so we did not report a mediation percentage; that analysis also assumes no unmeasured confounding, and rural residence and forest cover are derived from the same land cover map; the associations of green space with lower sensitisation should be interpreted cautiously given the cross-sectional, ecological exposure design. Sex was missing for 322 children and was therefore not included in the primary models.

We tested the robustness of these findings in several sensitivity analyses (Supplementary Tables S1–S5). Repeating the primary AIC comparison with a stricter sensitisation threshold (wheal ≥ 3 mm; n = 336 sensitised) and with continuous wheal diameter as the outcome gave essentially identical results, with PM_10_ and pollen area remaining informationally equivalent (ΔAIC 2.0 and 0.8, respectively); a continuous, untransformed PM_10_ exposure gave the same pattern (ΔAIC 0.5 versus pollen area), indicating that our conclusions do not depend on the specific outcome threshold or exposure dichotomisation used. Because PM_10_ takes only four distinct area-level values in this cohort, a median-split threshold produced an identical exposure classification to our primary 66th-percentile split. Region-stratified models showed a consistent protective direction for broadleaved forest and pasture cover in all three regions (Zagreb, Mediterranean, Slavonia), although individual strata were underpowered to reach significance at the smaller regional sample sizes; the pooled, region-adjusted estimates (broadleaved forest aOR 0.977 [95% CI 0.956–0.999], *p* = 0.041; pastures aOR 0.962 [95% CI 0.929–0.996], *p* = 0.027) are therefore likely to be more reliable than any single-region estimate. With standard errors clustered on school, the estimates for rural residence, total green area and pastures were similar, whereas the interval for broadleaved forest widened (Supplementary Table S5).

These findings have some practical implications. The similar sensitisation rates observed in Zagreb and Slavonia, despite a twofold difference in pollen concentrations, suggest that the relationship between pollen exposure and sensitisation may not be linear. This finding indicates that pollen-based alert systems may benefit from incorporating threshold effects. The observed association of surrounding green space with lower sensitisation suggests that access to natural vegetation may play a role in reducing sensitisation risk in children, though this hypothesis-generating finding would need confirmation in longitudinal studies before informing green-space policy directly. These findings tentatively support further investigation of green-space preservation and expansion as part of public health strategies [22,23,29]. Finally, indoor exposures contributed little to ragweed sensitisation in this cohort, which suggests that prevention research for this allergen should focus on the outdoor environment.

## 6. Conclusions

In this cross-sectional re-analysis of Croatian children, area-level particulate air pollution and pollen area were informationally equivalent predictors of ragweed sensitisation: both describe the contrast between the inland regions and the Mediterranean coast, and the available exposure data cannot distinguish which of them is the causal driver. This is consistent with the earlier analysis of this cohort by Agnew et al. [5], in which air pollutants were not significant once pollen area was accounted for, and it means that neither analysis can confirm or exclude a contribution of air pollution. We confirmed the association of rural residence with lower sensitisation reported by Agnew et al. [5] but could not determine whether broadleaved forest cover explains it. Although they found no association for categorised land cover, land cover on a continuous scale was nominally associated with lower sensitisation, transitional woodland-shrub after correction for multiple testing, and total green area and pastures. Indoor environmental exposures added little to the prediction of sensitisation, which is in agreement with the earlier analysis. Resolving the Zagreb paradox definitively will require cohorts with individual-level pollution measurements and sufficient spatial and temporal heterogeneity to disentangle highly collinear ecological predictors.

## Figures and Tables

**Table 1:** AIC model comparison for ragweed sensitisation (n = 3520; all models adjusted for age and parental atopy). “PM_10_ only” denotes a single-predictor model (PM_10_ binary, plus age and parental atopy). ΔAIC, difference from the best-fitting model; k, number of parameters. Models combining binary PM_10_ with pollen area are not listed because binary PM_10_ is fully determined by region.

Model	AIC	ΔAIC	k
PM_10_ only	2157.1	0.0	4
Pollen area (3-category)	2158.8	1.7	5
PM_10_ + NO_2_	2158.8	1.7	5
NO_2_ + Pollen area	2160.8	3.7	6
NO_2_ only	2367.9	210.8	4
Base (age + atopy)	2374.7	217.6	3

**Table 2:** Indoor variable prevalence by sensitisation status (n = 3520).

Variable	Sensitised (n = 380)	Non-Sensitised (n = 3140)	*p*
Environmental tobacco smoke	22.9%	26.9%	0.106
Pet ownership	81.1%	80.6%	0.877
Carpet	41.3%	41.9%	0.858
Feather bedding	32.6%	35.8%	0.246

**Table 3:** Four-model AIC comparison for indoor vs. outdoor predictors (n = 3520). Base: age + parental atopy. Indoor: environmental tobacco smoke + pet ownership + carpet + feather bedding, plus base covariates. Outdoor: PM_10_ (binary) + log-broadleaved forest + log-pastures + rural residence, plus base covariates. Combined: all outdoor and indoor terms plus base covariates.

Model	AIC	ΔAIC vs. Outdoor
Outdoor (PM_10_ + landscape)	2151.7	0.0
Combined (+indoor)	2150.5	−1.2
Base (age + atopy)	2374.7	+223.0
Indoor only	2377.4	+225.7

## Data Availability

The data presented in this study are available on request from the corresponding author, under specific conditions. The data is not publicly available due to ethical restrictions (sensitive data).

## Supplementary Materials

The following supporting information can be downloaded at https://www.mdpi.com/article/10.3390/children13101366/s1, Supplementary Methods: Sensitivity analyses, Supplementary Table S1. Alternative sensitisation definitions; Supplementary Table S1a. Primary outcome: wheal ≥ 2 mm (prevalence 10.8%); Supplementary Table S1b. Stricter outcome: wheal ≥ 3 mm (prevalence 9.5%; n = 336 sensitised); Supplementary Table S1c. Continuous outcome: wheal diameter, mm (linear regression; exploratory, zero-inflated outcome); Supplementary Table S2. Alternative PM_10_ exposure threshold and transformation; Supplementary Table S2a. Primary: PM_10_ ≥ 66th percentile (“high” in 60.1% of children); Supplementary Table S2b. Alternative: PM_10_ ≥ 50th percentile (median split; “high” in 60.1% of children, identical classification to S2a); Supplementary Table S2c. Alternative: PM_10_ and pollen as continuous (untransformed) exposures; Supplementary Table S3. Region-stratified landscape (green-space) models; Supplementary Table S4. Landscape screen: all sixteen variables; Supplementary Table S5. Rural residence, mediation and robustness analyses; Supplementary Table S6. Heating source and mattress age; Supplementary Table S7. Participant characteristics by study region.

## Abbreviations

The following abbreviations are used in this manuscript:

AIC	Akaike Information Criterion
aOR	adjusted odds ratio
ATOPICA	Atopic diseases in changing climate, land use and air quality
CI	Confidence interval
CORINE	Coordinated Information on the European Environment
EDIAQI	Evidence-driven indoor air quality improvement
EU	European Union
ETS	Environmental tobacco smoke
FP7	Framework programme 7
ISAAC	International Study of Asthma and Allergies in Childhood
NO_2_	Nitrogen dioxide
NO_x_	Nitric oxide (NO) and nitrogen dioxide (NO_2_)
O_3_	Ozone
PAH	Polycyclic aromatic hydrocarbons
PM_10_	Particulate matter with a diameter of 10 microns or less
SO_2_	Sulphur dioxide
SPT	Skin prick test
VOC	Volatile organic compounds
