# Supplementary material for: Outdoor Environmental Risk Factors for Paediatric Ragweed Sensitisation: A Formal Model Comparison
Source: Children (Basel). 2026 Oct 8;13(10):1366. doi: 10.3390/children13101366 (PMC13702990; doi:10.3390/children13101366)
Supplement: Supplementary file 1 [file children-13-01366-s001.zip › children-4560630-supplementary.pdf]

Supplementary Materials

Outdoor Environmental Risk Factors for Paediatric Ragweed Sensitisation: A Formal Model Comparison

Mario Lovrić, Ivana Banić, Kristina Časni, Iva Šunić, Ivana Jerković and Mirjana Turkalj

Supplementary Methods: Sensitivity analyses

To assess whether the primary findings depended on specific analytic choices, we repeated the AIC-based model comparison and the landscape (green-space) analysis under alternative outcome definitions, exposure thresholds and exposure transformations, stratified the landscape models by study region, and repeated the rural, landscape and joint models with standard errors clustered on school and with adjustment for sex. All analyses used the complete-case analytic sample of the main text (n = 3,520), except the school-clustered models (n = 3,431 with a recorded school) and the sex-adjusted models (n = 3,198 with recorded sex), and the same covariate adjustment (age, parental atopy) and the same modelling approach (binary logistic regression fitted by maximum likelihood; Python 3.11, statsmodels 0.14). Rural residence is the ATOPICA classification described in Section 2.3.3 of the main text.

Supplementary Table S1. Alternative sensitisation definitions

AIC comparison of pollen area versus air pollutants under three outcome definitions. All models adjusted for age and parental atopy; n = 3,520.

Supplementary Table S1a. Primary outcome: wheal ≥2 mm (prevalence 10.8%; n=380 sensitised)

Model	AIC	ΔAIC
PM10 only	2157.1	0.0
Pollen area (3-category)	2158.8	1.7
PM10 + NO2	2158.8	1.7
NO2 only	2367.9	210.8
Base (age + atopy)	2374.7	217.6

Supplementary Table S1b. Stricter outcome: wheal ≥3 mm (prevalence 9.5%; n=336 sensitised)

Model	AIC	ΔAIC
PM10 only	1978.3	0.0
PM10 + NO2	1980.2	1.9
Pollen area (3-category)	1980.3	2.0
NO2 only	2181.8	203.5
Base (age + atopy)	2184.4	206.1

Supplementary Table S1c. Continuous outcome: wheal diameter, mm (linear regression; exploratory, zero-inflated outcome)

Model	AIC	R^2	ΔAIC
PM10 only	13,733.4	0.0583	0.0
Pollen area (3-category)	13,734.2	0.0586	0.8
PM10 + NO2	13,735.2	0.0583	1.8
NO2 only	13,895.9	0.0138	162.5
Base (age + atopy)	13,905.0	0.0107	171.6

Interpretation: Under both a stricter sensitisation threshold and a continuous wheal-diameter outcome, PM10 and categorical pollen area remained informationally equivalent (ΔAIC 0.8 to 2.0), as in the primary analysis.

Supplementary Table S2. Alternative PM10 exposure threshold and transformation

Supplementary Table S2a. Primary: PM10 ≥66th percentile ("high" in 60.1% of children)

Model	AIC	ΔAIC
PM10 only	2157.1	0.0
Pollen area (3-category)	2158.8	1.7
PM10 + NO2	2158.8	1.7
Base (age + atopy)	2374.7	217.6

Supplementary Table S2b. Alternative: PM10 ≥50th percentile (median split; "high" in 60.1% of children, identical classification to S2a)

The median split classifies exactly the same children as the primary threshold, so every model in Table S2a is reproduced unchanged.

Supplementary Table S2c. Alternative: PM10 and pollen as continuous (untransformed) exposures

Model	AIC	ΔAIC
PM10 continuous	2158.3	0.0
Pollen area (3-category)	2158.8	0.5
PM10 continuous + NO2	2158.8	0.5
Pollen continuous	2266.1	107.8
Base (age + atopy)	2374.7	216.4

Interpretation: Area-level PM10 takes only four distinct values in this cohort (13.99, 15.81, 31.66, 32.16 µg/m³), two in the Mediterranean region and one each in Zagreb and Slavonia. Any cut-off between the Mediterranean and the inland values gives the same binary classification, which is identical to an inland versus Mediterranean indicator. Continuous PM10 reproduced the near-equivalence with pollen area (ΔAIC 0.5), whereas continuous pollen fitted much worse because Zagreb and Slavonia have the same sensitisation prevalence despite a twofold difference in pollen.

Supplementary Table S3. Region-stratified landscape (green-space) models

Association between log-transformed land cover area (5 km buffer) and ragweed sensitisation within each study region. Each model includes broadleaved forest, pastures, rural residence, age and parental atopy; the pooled model additionally includes study region.

Region	n	Variable	aOR	95% CI	p
Zagreb	1,142	Broadleaved forest	0.981	0.956-1.006	0.133
Zagreb	1,142	Pastures	0.944	0.897-0.994	0.028
Mediterranean	1,406	Broadleaved forest	0.980	0.913-1.052	0.571
Mediterranean	1,406	Pastures	0.986	0.908-1.070	0.731
Slavonia	972	Broadleaved forest	0.949	0.828-1.087	0.447
Slavonia	972	Pastures	0.970	0.900-1.046	0.428
**Pooled, region-adjusted**	**3,520**	**Broadleaved forest**	**0.977**	**0.956-0.999**	**0.041**
**Pooled, region-adjusted**	**3,520**	**Pastures**	**0.962**	**0.929-0.996**	**0.027**

Interpretation: Point estimates for broadleaved forest and pasture cover were below 1 in all three regions. Within regions only pastures in Zagreb reached nominal significance, which reflects the smaller samples and, in the Mediterranean region, the small number of sensitised children (n = 30).

Supplementary Table S4. Landscape screen: all sixteen variables

Each variable was entered separately in a logistic model adjusted for rural residence, study region, age and parental atopy (n = 3,520). Areas and distances are natural-log transformed (log of the value plus 1); the Shannon index is untransformed. Bonferroni-corrected threshold for 16 tests: $p < 0.003$.

Variable	Type	aOR per log-unit	95% CI	p	aOR per doubling
Transitional woodland-shrub	CORINE class (log area)	0.933	0.895-0.972	0.001 *	0.953
Total green area (composite)	composite (log area)	0.925	0.872-0.981	0.009	0.947
Pastures	CORINE class (log area)	0.963	0.930-0.998	0.039	0.974
Broadleaved forest	CORINE class (log area)	0.979	0.957-1.001	0.059	0.985
Discontinuous urban fabric	CORINE class (log area)	0.935	0.856-1.020	0.131	0.954
Urban area (composite)	composite (log area)	0.938	0.859-1.024	0.150	0.956
Road and rail networks	CORINE class (log area)	1.015	0.994-1.035	0.155	1.010
Distance to nearest motorway	distance (log)	1.074	0.953-1.211	0.244	1.051
Non-irrigated arable land	CORINE class (log area)	1.013	0.989-1.037	0.294	1.009
Mixed forest	CORINE class (log area)	1.009	0.985-1.033	0.481	1.006
Natural grasslands	CORINE class (log area)	1.017	0.968-1.068	0.503	1.012
Shannon diversity (composite)	composite (index)	0.886	0.558-1.405	0.606	n/a
Continuous urban fabric	CORINE class (log area)	1.004	0.986-1.023	0.644	1.003
Industrial or commercial units	CORINE class (log area)	1.006	0.980-1.032	0.660	1.004
Distance to nearest road	distance (log)	1.019	0.933-1.112	0.680	1.013
Coniferous forest	CORINE class (log area)	1.011	0.957-1.068	0.695	1.008

* below the Bonferroni-corrected threshold. Total green area is the summed area of broadleaved, coniferous and mixed forest, natural grasslands and pastures; including transitional woodland-shrub in the composite gave aOR 0.855 (95% CI 0.762-0.958; $p = 0.007$). Urban area is the summed area of continuous and discontinuous urban fabric, industrial or commercial units, and road and rail networks. The Shannon index is calculated across the 11 CORINE classes listed, as proportions of their summed area.

Share of children with zero area within 5 km: broadleaved forest 19%; coniferous forest 67%; mixed forest 80%; natural grasslands 84%; pastures 33%; transitional woodland-shrub 1%.

Supplementary Table S5. Rural residence, mediation and robustness analyses

Supplementary Table S5a. Rural residence and broadleaved forest cover

Model	Term	aOR	95% CI	p
Rural, region, age, parental atopy (n = 3,520)	Rural residence	0.69	0.50-0.96	0.026
+ broadleaved forest (n = 3,520)	Rural residence	0.72	0.52-0.99	0.046
+ broadleaved forest (n = 3,520)	Broadleaved forest	0.979	0.957-1.001	0.059
+ total green area (n = 3,520)	Rural residence	0.72	0.52-0.99	0.045
+ total green area (n = 3,520)	Total green area	0.925	0.872-0.981	0.009
Rural; SE clustered on school (n = 3,431)	Rural residence	0.68	0.52-0.88	0.004
+ broadleaved forest; SE clustered on school (n = 3,431)	Rural residence	0.70	0.54-0.92	0.010
+ broadleaved forest; SE clustered on school (n = 3,431)	Broadleaved forest	0.979	0.954-1.005	0.105
+ total green area; SE clustered on school (n = 3,431)	Rural residence	0.71	0.54-0.91	0.008
+ total green area; SE clustered on school (n = 3,431)	Total green area	0.919	0.859-0.983	0.014
Rural, additionally adjusted for sex (n = 3,198)	Rural residence	0.68	0.49-0.94	0.021
Rural, additionally adjusted for sex (n = 3,198)	Boys vs girls	1.70	1.35-2.14	<0.001

School was recorded for 3,431 children (105 schools, of which 53 included rural children) and sex for 3,198 children. Clustered intervals for rural residence are not wider than the model-based ones and should not be read as stronger evidence.

Supplementary Table S5b. Counterfactual mediation analysis: rural residence, broadleaved forest cover and sensitisation

Mediator model: linear regression of log broadleaved forest area on rural residence. Outcome model: logistic regression of sensitisation on rural residence, log broadleaved forest area and their interaction. Both adjusted for study region, age and parental atopy (n = 3,520). For each child the mediator was set to its model-predicted mean under rural and under urban residence (no random draw), and predicted risks were averaged over children. Effects are risk differences in percentage points; 95% CIs are percentiles of 2,000 nonparametric bootstrap replicates.

Quantity	Estimate	95% CI
Total effect of rural residence	-3.07 pp	-5.41 to -0.47
Natural direct effect	-1.42 pp	-6.82 to 2.77
Natural indirect effect (through forest cover)	-1.65 pp	-4.50 to 3.25
Proportion mediated	53.86%	-164.69 to 357.62

Interpretation: Rural residence was associated with a lower risk of sensitisation, but under the assumption of no unmeasured confounding the data could not separate a direct effect from an effect through broadleaved forest cover, and the proportion mediated is not estimable with useful precision.

Supplementary Table S5c. Joint outdoor model with model-based and school-clustered standard errors

Model	Term	aOR	95% CI	p
Joint outdoor model (n = 3,520)	PM10 (high vs low)	16.08	9.10-28.40	<0.001
Joint outdoor model (n = 3,520)	Broadleaved forest	0.982	0.961-1.003	0.091
Joint outdoor model (n = 3,520)	Pastures	0.963	0.930-0.997	0.035
Joint outdoor model (n = 3,520)	Rural residence	0.72	0.52-1.00	0.051
Joint outdoor model; SE clustered on school (n = 3,431)	PM10 (high vs low)	16.47	8.44-32.13	<0.001
Joint outdoor model; SE clustered on school (n = 3,431)	Broadleaved forest	0.982	0.959-1.007	0.152
Joint outdoor model; SE clustered on school (n = 3,431)	Pastures	0.962	0.927-0.998	0.040
Joint outdoor model; SE clustered on school (n = 3,431)	Rural residence	0.71	0.55-0.93	0.014
Joint outdoor model + sex (n = 3,198)	PM10 (high vs low)	19.40	9.50-39.61	<0.001
Joint outdoor model + sex (n = 3,198)	Broadleaved forest	0.982	0.960-1.004	0.112
Joint outdoor model + sex (n = 3,198)	Pastures	0.953	0.916-0.992	0.018
Joint outdoor model + sex (n = 3,198)	Rural residence	0.70	0.50-0.98	0.035
Joint outdoor model + sex (n = 3,198)	Boys vs girls	1.71	1.36-2.16	<0.001

Supplementary Table S6. Heating source and mattress age (exploratory)

Each variable entered separately, adjusted for age and parental atopy, and additionally for study region. Heating type differs strongly between regions (electric heating in 74% of Mediterranean and 4% of inland homes), so the region-adjusted column is the relevant one.

Variable	n	Exposed	aOR (age, parental atopy)	95% CI	p	aOR (+ study region)	95% CI	p
Gas heating	3,510	22.6%	1.80	1.43-2.28	<0.001	0.96	0.76-1.23	0.762
Oil heating	3,510	1.5%	0.85	0.33-2.15	0.725	0.77	0.30-1.99	0.584
Electric heating	3,510	31.7%	0.16	0.11-0.24	<0.001	0.68	0.41-1.13	0.135
Wood heating	3,510	23.4%	0.77	0.59-1.00	0.053	0.75	0.57-0.99	0.043

Central heating	3,509	42.1%	1.95	1.57-2.42	<0.001	1.20	0.96-1.51	0.113
Mattress older than 3 years, any period	3,469	53.4%	0.81	0.65-1.01	0.058	0.76	0.61-0.96	0.020
Mattress older than 3 years, first year of life	3,441	12.7%	0.81	0.58-1.14	0.234	0.80	0.56-1.14	0.219
Mattress older than 3 years, age 2 to 7	3,383	35.4%	0.89	0.70-1.12	0.303	0.84	0.67-1.07	0.163
Mattress older than 3 years, after age 7	1,928	54.7%	0.83	0.63-1.09	0.173	0.82	0.62-1.08	0.148

Supplementary Table S7. Participant characteristics by study region (analytic sample, n = 3,520)

Characteristic	Zagreb	Mediterranean	Slavonia	All
Children, n	1,142	1,406	972	3,520
Ragweed-sensitised, n (%)	187 (16.4)	30 (2.1)	163 (16.8)	380 (10.8)
Age, years, mean (SD)	7.1 (1.8)	6.9 (1.8)	7.0 (1.7)	7.0 (1.8)
Boys, % of those with recorded sex (n)	51.1 (1,142)	48.2 (1,084)	54.2 (972)	51.1 (3,198)
Parental atopy, %	39.6	39.5	34.7	38.2
Rural residence, n (%)	143 (12.5)	65 (4.6)	232 (23.9)	440 (12.5)
Environmental tobacco smoke, %	25.0	24.9	30.6	26.5
Pet ownership, %	82.5	77.5	82.9	80.6
Carpet, %	43.2	36.7	47.8	41.9
Feather bedding, %	33.2	35.0	38.8	35.5
Schools, n	39	37	29	105

Area-level exposures take four values each. PM10: 32.16 µg/m³ (Zagreb), 31.66 (Slavonia), and 15.81 and 13.99 in the two Mediterranean areas (1,084 and 322 children). NO2: 32.79 µg/m³ (Zagreb), 18.46 (Slavonia), and 28.88 and 16.74 in the same two Mediterranean areas.
